# Organocatalytic asymmetric fluorination of α-chloroaldehydes involving kinetic resolution

**DOI:** 10.3762/bjoc.10.30

**Published:** 2014-02-04

**Authors:** Kazutaka Shibatomi, Takuya Okimi, Yoshiyuki Abe, Akira Narayama, Nami Nakamura, Seiji Iwasa

**Affiliations:** 1 Department of Environmental and Life Sciences, Toyohashi University of Technology, 1-1 Hibarigaoka, Tempaku-cho, Toyohashi 441-8580, Japan

**Keywords:** α-branched aldehyde, asymmetric catalysis, chlorination, fluorination, organocatalyst, organo-fluorine

## Abstract

In a previous study it was shown that the enantioselective α-fluorination of racemic α-chloroaldehydes with a chiral organocatalyst yielded the corresponding α-chloro-α-fluoroaldehydes with high enantioselectivity. It was also revealed that kinetic resolution of the starting aldehydes was involved in this asymmetric fluorination. This paper describes the determination of the absolute stereochemistry of a resulting α-chloro-α-fluoroaldehyde. Some information about the substrate scope and a possible reaction mechanism are also described which shed more light on the nature of this asymmetric fluorination reaction.

## Introduction

Fluorinated organic molecules are of considerable interest in pharmaceutical and agricultural chemistry owing to the unique properties of the fluorine atom [1–2]. These compounds, especially with one or more fluorinated stereogenic center(s), are fascinating building blocks for new drug candidates. Organocatalytic α-fluorination of aldehydes is known to be an efficient strategy for the enantioselective construction of fluorinated chiral carbon centers [3–6]; however, very few successful studies have been published on the fluorination of α-branched aldehydes [7]. During the course of our study on the enantioselective construction of such fluorinated stereogenic centers, we developed a method for the enantioselective synthesis of α-chloro-α-fluoroaldehydes via the organocatalytic α-fluorination of α-alkyl-α-chloroaldehydes, a type of α-branched aldehyde, mediated by the Jørgensen–Hayashi catalyst **1** [8]. The reaction yielded the desired α-chloro-α-fluoroaldehydes with high enantioselectivity when the starting aldehyde was used in excess over *N*-fluorobenzenesulfonimide (NFSI) in the reaction. However, when an excess NFSI with respect to the starting aldehyde was used, poor asymmetric induction was observed. In this paper, we describe the determination of the absolute stereochemistry of a resulting α-chloro-α-fluoroaldehyde using this methodology and discuss the possible reaction mechanism that involves kinetic resolution.

## Results and Discussion

**Scheme 1:**
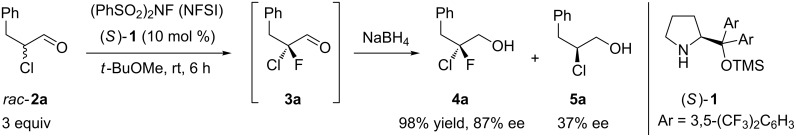
Organocatalytic enantioselective fluorination of α-chloroaldehyde **2a** [8].

In our previous study [8], enantioselective fluorination of racemic 2-chloro-3-phenylpropanal (**2a**) was carried out with 3 equiv of NFSI in the presence of organocatalyst (*S*)-**1** to yield the corresponding α-chloro-α-fluoroaldehyde **3a** in good conversion. Isolation of the product and determination of enantiomeric purity were performed after reduction to primary alcohol **4a** because **3a** was unstable to silica gel chromatography. The reaction afforded **4a** with high enantioselectivity along with the monochloro alcohol **5a**, whose enantiomeric purity was determined to be 37% ee (Scheme 1) [8]. These results suggested that kinetic resolution of the starting aldehydes was involved in this asymmetric fluorination.

**Scheme 2:**
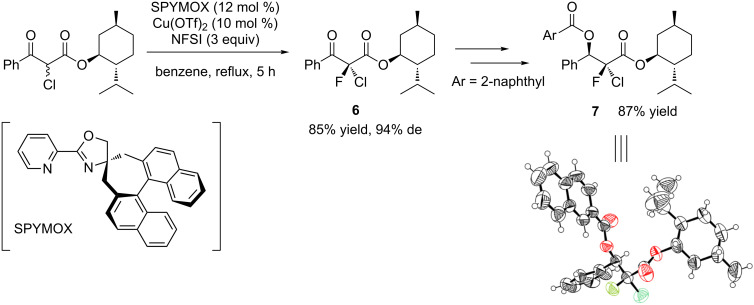
Determination of absolute configuration of α-chloro-α-fluoro-β-keto ester **6** by X-ray analysis [9].

**Scheme 3:**
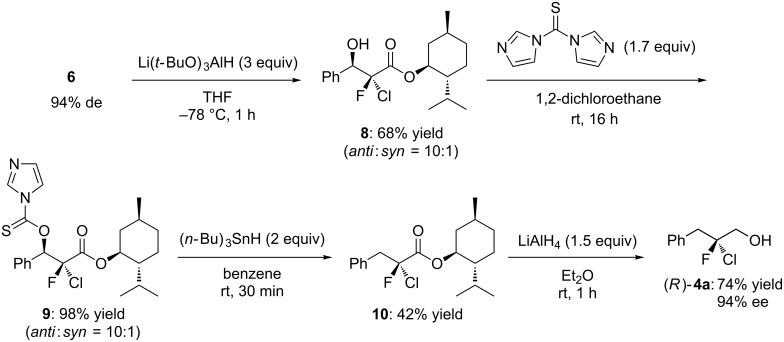
Transformation of α-chloro-α-fluoro-β-keto ester **6** to chlorofluoro alcohol **4a**.

To collect further information on the reaction mechanism, we sought to determine the absolute configuration of **4a**. Recently, we reported the enantioselective synthesis of α-chloro-α-fluoro-β-keto esters via the sequential chlorination–fluorination of β-keto esters with the Cu(II) complex of SPYMOX [9], a spiro chiral oxazoline ligand developed by our research group [9–12]. In that study, we succeeded in determining the absolute stereochemistry of the α-chloro-α-fluoro-β-keto ester **6** by the X-ray crystallographic analysis of its derivative **7** (Scheme 2). Here, our aim was to transform chlorofluoro ester **6** to **4a** in order to compare its optical rotation with that of **4a** derived from **2a** in the presence of catalyst (*S*)-**1**. As shown in Scheme 3, β-keto ester **6** was converted via the Barton–McCombie deoxygenation [13] into a simple ester **10**, which was then reduced to the primary alcohol **4a** by treatment with LiAlH_4_. Comparison of the optical rotations and retention times on chiral HPLC clearly showed that the asymmetric fluorination of **2a** catalyzed by (*S*)-**1** yielded **4a** having the *R* configuration (Scheme 1).

An investigation of the substrate scope of the organocatalytic fluorination of α-chloroaldehydes was performed as shown in Table 1. The reaction of **2a** with 3 equiv of NFSI yielded **4a** in 87% ee along with monochloro alcohol **5a** in 37% ee (Table 1, entry 2) as described above. On the other hand, the reaction with 2 equiv of NFSI against to **2a** showed poor enantioselectivity (31% ee, Table 1, entry 1). We also examined the reaction with 2 equiv of **2a** based on NFSI. The reaction yielded **4a** in 75% ee (lower ee than that in Table 1, entry 2), and the enantiomeric purity of the recovered **5a** was increased to 52% ee (Table 1, entry 3). Similar trends were observed in the fluorination with some other substrates **2b**–**2g** (Table 1, entries 4–14). These results strongly suggested that the high asymmetric induction in this fluorination requires not only control of enantiofacial selection during electrophilic fluorination of the enamine intermediates, but also a high level of kinetic resolution of the starting aldehydes.

**Table 1:** Enantioselective fluorination of α-chloroaldehydes.^a^

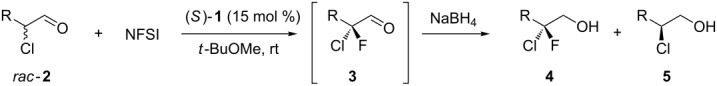						

entry	R	**2**:NFSI	*t* (h)	% yield of **4**^b^	% ee of **4**^c^	% ee of **5**^c,d^

1^e^	Bn (**2a**)	1:2	11	78	31 (*R*)	–
2^e,f^	Bn	3:1	6	98	87 (*R*)	37 (*S*)
3	Bn	2:1	6	96	75 (*R*)	52 (*S*)
4	*n*-Hex (**2b**)	1:2	11	82	31	–
5^e,f^	*n*-Hex	3:1	10	97	80	35 (*S*)
6	*n*-Hex	2:1	19	92	68	49 (*S*)
7	–(CH_2_)_3_OCH_2_OCH_3_ (**2c**)	1:2	19	83	23	–
8^f^	–(CH_2_)_3_OCH_2_OCH_3_	3:1	10	90	78	33 (*S*)
9^f^	–(CH_2_)_3_CO_2_Et (**2d**)	3:1	4	90	80	20
10^g^	*c*-Hex (**2e**)	1:2	48	88	42	–
11^g^	*c*-Hex	3:1	24	92	96	15
12	Ph (**2f**)	1:2	12	61	72	–
13^e,f^	Ph	3:1	10	82	90	5
14^e,g^	*t*-Bu (**2g**)	3:1	30	87	99	29

^a^Reactions were carried out in *t*-BuOMe with 15 mol % of (*S*)-**1** unless otherwise noted. ^b^Isolated yield based on **2** or NFSI. ^c^Determined by chiral HPLC or GC analysis. ^d^Monochloro alcohol **5** was recovered in nearly quantitative yield. ^e^Similar result was reported in Ref. [8]. ^f^10 mol % of (*S*)-**1** was used. ^g^Reaction was carried out with 30 mol % of (*S*)-**1** at 30 °C.

**Scheme 4:**
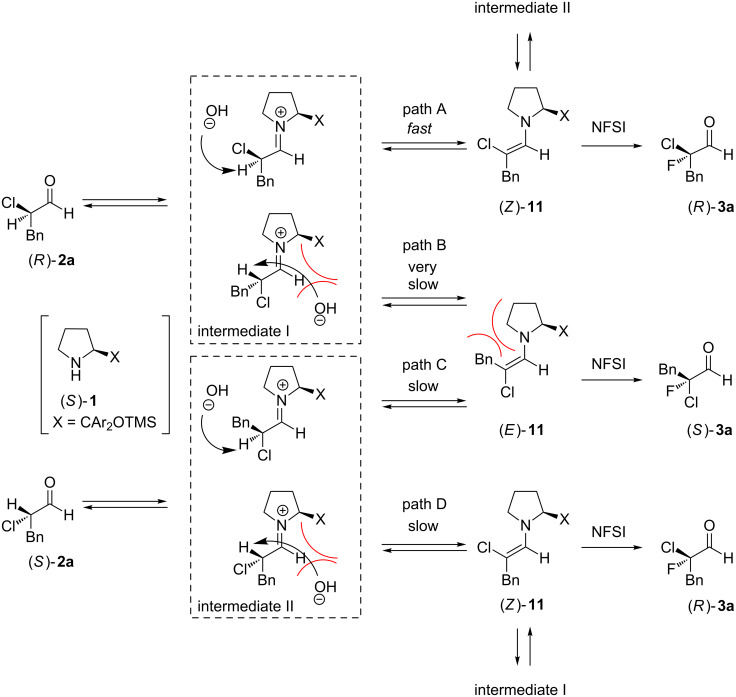
Proposed reaction mechanism.

From these results, we proposed a reaction mechanism for the fluorination of α-chloroaldehydes, as shown in Scheme 4. Catalyst (*S*)-**1** reacts with (*R*)-**2a** to form iminium intermediate I, which undergoes deprotonation from the side opposite to the bulky substituent X (X = CAr_2_OTMS) of the pyrrolidine ring to afford enamine intermediate (*Z*)-**11** (path A). Then, NFSI attacks (*Z*)-**11** from the side opposite to X to yield (*R*)-**3a**. Although deprotonation may also occur from the same side as X to give (*E*)-**11** (path B), the reaction through path B is considered to be very slow because the steric repulsion between the counter anion (OH^–^) and X would prevent deprotonation. Further, the resulting (*E*)-**11** would be a thermodynamically unfavorable product because of steric repulsion between the methylene group on the pyrrolidine ring and the benzyl substituent on **2a**. Alternatively, (*S*)-**2a** reacts with (*S*)-**1** to form iminium intermediate II, which also undergoes deprotonation to form (*E*)- or (*Z*)-**11**. In these cases, deprotonation from the side opposite to X (path C) is considered to be slow because the resulting (*E*)-**11** is a thermodynamically unfavorable form, as described above, and deprotonation from the same side as X (path D) is also slow because of steric repulsion between the counter anion (OH^–^) and X. Thus, it is difficult to control the geometry of enamine intermediate **11** when starting from (*S*)-**2a**, and hence, the enantioselectivity of the fluorination is significantly decreased because the fluorination occurs from the side opposite to X, regardless of the geometry of **11**. For these reasons, high enantioselectivity was observed when **2a** was employed in excess in the reaction, whereas an excess of NFSI led to poor asymmetric induction. In the former reaction, the major enantiomer of the recovered **5a** was the *S*-form (Table 1, entries 2 and 3). This result also supports the proposed mechanism.

**Scheme 5:**
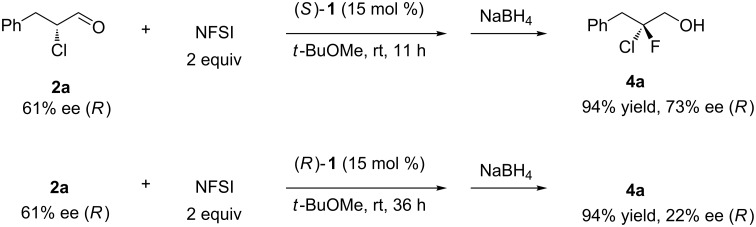
Fluorination of the enantiomers of **2a**.

To test the proposed reaction mechanism, we carried out the fluorination of enantioenriched **2a** (61% ee, *R* favored) with 2 equiv of NFSI in the presence of each enantiomer of catalyst **1**. As expected from the mechanism, good enantioselectivity was observed when (*S*)-**1** was employed in the reaction, whereas the reaction proceeded more slowly to yield **4a** with poor enantioselectivity in the presence of (*R*)-**1** (Scheme 5).

**Scheme 6:**
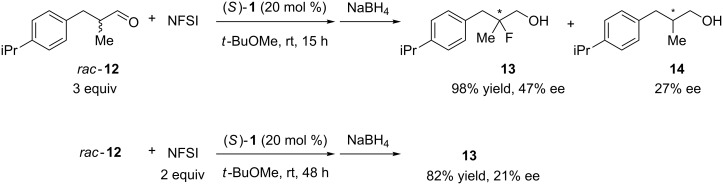
Enantioselective fluorination of α-branched aldehyde **12**.

Finally, we were curious to know whether a similar kinetic resolution would be observed in the fluorination of α,α-dialkylaldehydes. We examined the fluorination of racemic α,α-dialkylaldehyde **12** in the presence of catalyst **1** (Scheme 6). The reaction with 3 equiv of *rac*-**12** based on NFSI afforded the corresponding product **13** in higher enantioselectivity than that obtained in the reaction with 2 equiv of NFSI, along with 27% ee of **14**; however the enantiomeric excess of **13** was not sufficiently high (47% ee). These results suggested that the reaction proceeded by a similar mechanism as shown in Scheme 4.

## Conclusion

In conclusion, we succeeded in the highly enantioselective fluorination of α-chloroaldehydes to afford α-chloro-α-fluoroaldehydes mediated by chiral organocatalyst **1**. It was revealed that kinetic resolution of the racemic α-chloroaldehydes occurred during this fluorination reaction, which played an important role in the asymmetric induction.

## Experimental

Experiments involving moisture- and/or air-sensitive compounds were performed in oven-dried flasks under an atmosphere of dry argon. All reactions were magnetically stirred and monitored by thin-layer chromatography (TLC) using pre-coated silica gel plates with F_254_ indicator. Visualization was accomplished with UV light (254 nm), or phosphomolybdic acid, potassium permanganate, or anisaldehyde staining. Column chromatography was performed over silica gel (40–100 μm). ^1^H, ^13^C, and ^19^F NMR spectra were acquired on a JEOL JNM-ECX500 spectrometer. Chemical shift values (δ) are reported in ppm (^1^H: δ 0.00 for tetramethylsilane; ^19^F: δ 0.00 for trichlorofluoromethane; ^13^C: δ 77.0 for residual chloroform). IR spectra were measured on a JASCO FT/IR-230 spectrometer. Elemental analysis was performed with a Yanaco CHN CORDER MT-6. High-performance liquid chromatography (HPLC) analyses were performed with a JASCO PU-1586 with a UV-1575 UV–vis detector using a chiral column. GC analysis was performed with a Shimadzu model 2014 instrument. Optical rotations were measured on a JASCO P-1030 polarimeter.

α-Chloro aldehydes **2** were prepared with *N*-chlorosuccinimide in the presence of organocatalyst according to the procedure reported by Jørgensen [14] and were distilled before use. Racemic forms were synthesized with DL-proline catalyst, and optically active **2a** was synthesized with L-prolinamide catayst, whose enantiopurity was slightly decreased during the distillation.

We confirmed that the optical purity of fluorinated products **4** did not change even after chromatographic purification using achiral silica gel and subsequent solvent evaporation. Therefore, we concluded that the enantiomers did not undergo self-disproportionation during the purification process [15–19].

### Transformation of **6** to (*R*)-**4a**

Compound **8** was synthesized from **6** (94% de) according to the procedure reported in [9]. A flame-dried flask under argon was charged with **8** (*anti*/*syn* = 10:1, 0.35 mmol) and 1,2-dichloroethane (2 mL). 1,1′-Thiocarbonyldiimidazole (0.6 mmol) was added to this solution, and the mixture was stirred for 16 h at ambient temperature. The mixture was quenched by adding saturated aqueous NaHCO_3_ and extracted with CH_2_Cl_2_. The organic layer was dried over Na_2_SO_4_ and concentrated under reduced pressure. The crude mixture was purified by silica gel column chromatography (1:1 hexane/Et_2_O) to give **9** in 98% yield (*anti*/*syn* = 10:1).

**9**: ^1^H NMR (400 MHz, CDCl_3_) δ 8.33 (s, 1H), 7.83–7.72 (m, 6H), 7.06 (s, 1H), 6.74 (d, *J* = 21.6 Hz, 1H), 4.87 (td, *J* = 10.8, 4.4 Hz, 1H), 2.01–1.94 (m, 1H), 1.74–1.56 (m, 4H), 1.55–1.36 (m, 2H), 1.18–0.96 (m, 2H), 0.92 (d, *J* = 6.4 Hz, 3H), 0.74 (d, *J* = 7.2 Hz, 3H), 0.52 (d, *J* = 7.2 Hz, 3H); ^13^C NMR (100 MHz, CDCl_3_) δ 180.6, 163.6 (d, *J* = 29.4 Hz), 137.1, 131.4, 130.3, 129.9, 129.8, 128.8, 128.6, 117.8, 103.3 (d, *J* = 262.8 Hz), 84.5 (d, *J* = 19.8 Hz), 78.8, 46.8, 40.1, 33.9, 31.5, 26.2, 22.8, 22.0, 20.7, 15.2; ^19^F NMR (376 MHz, CDCl_3_) δ −132.1 (d, *J* = 21.4 Hz); FTIR (neat) υ_max_: 2955, 1762, 1464, 1395, 1288, 1212, 1102, 992, 952, 742, 475 cm^−1^; anal calcd (%) for C_23_H_28_ClFN_2_O_3_S: C, 59.15; H, 6.04; N, 6.00; found: C, 59.18; H, 5.96; N, 6.40.

A flame-dried flask under argon was charged with **9** (0.22 mmol) and benzene (3.6 mL). Tributyltin hydride (0.45 mmol) was added to this solution, and the mixture was stirred for 30 min at room temperature. The solvent was removed under reduced pressure and the crude mixture was purified by silica gel column chromatography (5:1 hexane/CH_2_Cl_2_) to give **10** in 42% yield.

**10**: ^1^H NMR (400 MHz, CDCl_3_) δ 7.35–7.25 (m, 5H), 4.74 (td, *J =* 10.8, 4.4 Hz, 1H), 3.65 (d, *J =* 6.8 Hz, 1H), 3.59 (d, *J =* 3.2 Hz, 1H), 1.85–1.76 (m, 2H), 1.72–1.63 (m, 2H), 1.50–1.41 (m, 2H), 1.37–1.19 (m, 1H), 1.09–0.98 (m, 1H), 0.95–0.91 (m, 1H), 0.89 (d, *J =* 2.4 Hz, 3H), 0.87 (dd, *J =* 3.2 Hz, 3H), 0.74 (dd, *J =* 7.2 Hz, 3H); ^13^C NMR (100 MHz, CDCl_3_) δ 165.3 (d, *J* = 27.4 Hz), 132.3, 130.7, 130.6, 128.5, 127.9, 106.1 (d, *J* = 257.9 Hz), 77.9, 46.8, 40.0, 34.0, 31.4, 26.1, 23.3, 22.0, 20.8, 16.1; ^19^F NMR (376 MHz, CDCl_3_) δ −116.6 (dd, *J =* 22.9, 19.6 Hz); FTIR (neat) υ_max_: 2954, 1754, 1458, 1282, 1216, 1145, 1043, 952, 704, 624, 471 cm^−1^; anal calcd (%) for C_19_H_26_ClFO_2_: C, 66.95; H, 7.69; found: C, 67.02; H, 7.96.

A flame-dried flask under argon was charged with **10** (0.07 mmol) and Et_2_O (0.2 mL). LiAlH_4_ (0.11 mmol) was added to this solution at −78 °C, and the mixture was stirred for 1 h at room temperature. The reaction mixture was quenched with saturated aqueous NH_4_Cl and the mixture was extracted with Et_2_O. The organic layer was dried over Na_2_SO_4_ and concentrated under reduced pressure. The crude mixture was purified by silica gel column chromatography (5:1 hexane/EtOAc) to give (*R*)-**4a** in 74% yield, with an enantiomeric purity of 94% ee.

**4a**: [α]_D_ = −2.8 (*c* 1.5, CHCl_3_). HPLC (99:1 hexane/2-propanol; 1 mL/min; using a CHIRALPAK IC column (0.46 cm Ø × 25 cm)): 11.4 min (major) and 11.9 min (minor). These analytical data were identical to those of **4a** synthesized from **2a** with (*S*)-**1**.

### General procedure for the asymmetric fluorination of α-chloroaldehydes **2**

To a solution of α-chloroaldehyde **2** (1.5 mmol) in *t-*BuOMe (2 mL) was added catalyst **1** (0.05 mmol) and NFSI (0.5 mmol). The reaction mixture was stirred at room temperature for the time given in Table 1 and then poured into MeOH/CH_2_Cl_2_ (1:4, 5 mL) at 0 °C. To this solution, NaBH_4_ (5 mmol) was added, and the mixture was stirred at room temperature for 1 h. The reaction was quenched with saturated aqueous NH_4_Cl, and the mixture was extracted with Et_2_O. The organic layer was dried over Na_2_SO_4_, concentrated, and chromatographed on silica gel to give **4**, along with monochloro alcohol **5**.

The results of all spectroscopic analyses of compounds **4a**, **4b**, **4f**, **4g**, and **5a–5f** were identical to those described in our previous report [8] and in references [20–21]. Absolute configuration of **5a**–**5c** was confirmed by comparing their optical rotation to that reported in the above-mentioned literature [20].

**(*R*)-2-Chloro-2-fluoro-3-phenylpropan-1-ol (4a, 87% ee)**: ^1^H NMR (500 MHz, CDCl_3_) δ 7.35–7.30 (m, 5H), 3.88–3.71 (m, 2H), 3.46 (dd, *J* = 32.3, 15.0 Hz, 2H), 2.15 (br, 1H); ^13^C NMR (125 MHz, CDCl_3_) δ 133.3 (d, *J* = 3.8 Hz), 130.7, 128.4, 127.6, 114.8 (d, *J* = 247 Hz), 67.2 (d, *J* = 26.4 Hz), 44.6 (d, *J* = 21.4 Hz); ^19^F NMR (470 MHz, CDCl_3_) δ −114.2 (m); [α]_D_ = −2.7 (*c* 1.5, CHCl_3_). The enantiopurity was determined by HPLC (99:1 hexane/2-propanol; 1 mL/min; using a CHIRALPAK IC column (0.46 cm Ø × 25 cm)): 11.4 min (major) and 11.9 min (minor).

**2-Chloro-2-fluorooctan-1-ol (4b, 80% ee)**: ^1^H NMR (500 MHz, CDCl_3_) δ 3.91–3.78 (m, 2H), 2.14–2.05 (m, 3H), 1.59–1.54 (m, 2H), 1.37–1.29 (m, 6H), 0.90 (t, *J* = 7.0 Hz, 3H); ^13^C NMR (125 MHz, CDCl_3_) δ 116.1 (d, *J* = 245 Hz), 68.3 (d, *J* = 26.4 Hz), 38.5 (d, *J* = 21.3 Hz), 31.5, 28.9, 23.3 (d, *J* = 3.8 Hz), 22.5, 14.0; ^19^F NMR (470 MHz, CDCl_3_) δ −113.9 (br); [α]_D_ = +2.0 (*c* 0.5, CHCl_3_). The enantiopurity was determined by GC (100–150 °C, 3 °C/min; using a Chiral DEX B-DM column): 12.4 min (major) and 13.3 min (minor).

**2-Chloro-2-fluoro-5-(methoxymethoxy)pentan-1-ol (4c, 78% ee)**: ^1^H NMR (500 MHz, CDCl_3_) δ 4.63 (s, 2H), 3.99–3.79 (m, 2H), 3.66–3.55 (m, 2H), 3.37 (s, 3H), 2.40–2.12 (m, 3H), 2.01–1.82 (m, 2H); ^13^C NMR (125 MHz, CDCl_3_) δ 115.8 (d, *J* = 245 Hz), 96.4, 68.4 (d, *J* = 26.4 Hz), 66.8, 55.3, 35.4 (d, *J* = 21.6 Hz), 23.9 (d, *J* = 4.8 Hz); ^19^F NMR (470 MHz, CDCl_3_) δ −114.3 (m); [α]_D_^22^ = +4.6 (*c* 0.16, CHCl_3_); anal calcd (%) for C_7_H_14_ClFO_3_: C, 41.91; H, 7.03; Cl, 17.67; F, 9.47; O, 23.92; found: C, 44.91; H, 7.51. The enantiopurity was determined after conversion into the corresponding 2-naphthoate **15c**.

A flame-dried flask under argon was charged with **4c** (0.10 mmol) and CH_2_Cl_2_ (1.0 mL). Triethylamine (0.20 mmol), 2-naphthoyl chloride (0.15 mmol), and 4-dimethylaminopyridine (0.01 mmol) were added to this solution, and the mixture was stirred for 2 h at 0 °C. The mixture was diluted by saturated aqueous NaHCO_3_, and extracted with CH_2_Cl_2_. The organic layer was dried over Na_2_SO_4_ and concentrated under reduced pressure. The crude mixture was purified by silica gel column chromatography (hexane/ethyl acetate 5:1) to give the desired 2-naphthoate **15c** in 82% yield.

**2-Chloro-2-fluoro-5-(methoxymethoxy)pentyl 2-naphthoate (15c, 78% ee)**: ^1^H NMR (400 MHz, CDCl_3_) δ 8.65 (s, 1H), 8.08 (d, *J* = 10.4 Hz, 1H), 7.98 (d, *J* = 8.4 Hz, 1H), 7.93–7.86 (m, 2H), 7.67–7.53 (m, 2H), 4.83–4.66 (m, 2H), 4.61 (s, 2H), 3.62 (t, *J* = 5.80 Hz, 2 H), 3.34 (s, 3H), 2.49–2.19 (m, 2H), 2.11–1.88 (m, 2H); ^13^C NMR (100 MHz, CDCl_3_) δ 165.6, 135.8, 132.4, 131.6, 129.4, 128.6, 128.4, 127.8, 126.8, 126.3, 125.2, 112.8 (d, *J* = 247 Hz), 96.4, 68.1 (d, *J* = 26.8 Hz), 66.6, 55.2, 36.2 (d, *J* = 22.0 Hz), 23.9 (d, *J* = 3.83 Hz); ^19^F NMR (470 MHz, CDCl_3_) δ −111.7 (m); [α]_D_^22^ = +7.5 (*c* 0.36, CHCl_3_); anal calcd (%) for C_18_H_20_ClFO_4_: C, 60.93; H, 5.68; Cl, 9.99; F, 5.35; O, 18.04; found: C, 60.95; H, 5.85. The enantiopurity was determined by HPLC (50:1 hexane/2-propanol; 0.5 mL/min; using a CHIRALPAK ID column (0.46 cm Ø × 25 cm)): 25.1 min (major) and 30.5 min (minor).

**Ethyl 5-chloro-5-fluoro-6-hydroxyhexanoate (4d, 80% ee)**: ^1^H NMR (500 MHz, CDCl_3_) δ 4.14 (q, *J* = 7.3 Hz, 2H), 3.94–3.80 (m, 2H), 2.58 (s, 1H), 2.44–2.34 (m, 2H), 2.28–2.07 (m, 2H), 1.95–1.86 (m, 2H), 1.26 (t, *J* = 7.3 Hz, 3H); ^13^C NMR (125 MHz, CDCl_3_) δ 173.1, 115.4 (d, *J* = 246.5 Hz), 68.1 (d, *J* = 26.5 Hz), 60.6, 37.4 (d, *J* = 22.8 Hz), 33.3, 18.9 (d, *J* = 4.8 Hz), 14.2; ^19^F NMR (470 MHz, CDCl_3_) δ −114.0 (m); [α]_D_^13^ = −1.48 (*c* 1.1, CHCl_3_); anal calcd (%) for C_8_H_14_ClFO_3_: C, 45.19; H, 6.64; found: C, 44.65; H, 6.67. The enantiopurity was determined after conversion into the corresponding 2-naphthoate **15d** by a procedure similar to that employed for the synthesis of **15c**. The crude mixture was purified by silica gel column chromatography (hexane/EtOAc = 10:1) to give 81% yield of **15d**.

**2-Chloro-6-ethoxy-2-fluoro-6-oxohexyl 2-naphthoate (15d, 80% ee)**: ^1^H NMR (500 MHz, CDCl_3_) δ 8.65 (s, 1H), 8.07 (d, *J* = 8.8 Hz, 1H), 7.99 (d, *J* = 8.0 Hz, 1H), 7.92–7.89 (m, 2H), 7.64–7.55 (m, 2H), 4.79–4.66 (m, 2H), 4.13 (q, *J* = 7.0 Hz, 2H), 2.48–2.38 (m, 2H), 2.37–2.17 (m, 2H), 2.09–1.95 (m, 2H), 1.24 (t, *J* = 7.0 Hz, 3H); ^13^C NMR (125 MHz, CDCl_3_) δ 172.7, 165.6, 135.7, 132.4, 131.6, 129.5, 128.6, 128.4, 127.8, 126.8, 126.2, 125.1, 112.5 (d, *J* = 247.6 Hz), 67.9 (d, *J* = 27.6 Hz), 60.5, 38.4 (d, *J* = 22.8 Hz), 33.4, 18.9 (d, *J* = 4.8 Hz), 14.2; ^19^F NMR (470 MHz, CDCl_3_) δ −111.8 (m); [α]_D_^21^ = +7.1 (*c* 0.31, CHCl_3_); anal calcd (%) for C_19_H_20_ClFO_4_: C, 62.21; H, 5.50; found: C, 62.92; H, 6.07. The enantiopurity was determined by HPLC (50:1 hexane/2-propanol; 1.0 mL/min; using a CHIRALPAK IB-3 column (0.46 cm Ø × 25 cm)): 19.5 min (minor) and 24.9 min (major).

**2-Chloro-2-cyclohexyl-2-fluoroethan-1-ol (4e, 96% ee)**: ^1^H NMR (500 MHz, CDCl_3_) δ 4.02–3.83 (m, 2H), 2.19–2.08 (m, 1H), 1.98–1.92 (m, 1H), 1.89–1.78 (m, 3H), 1.74–1.66 (m, 1H), 1.39–1.11 (m, 6H); ^13^C NMR (125 MHz, CDCl_3_) δ 119.0 (d, *J* = 247 Hz), 66.8 (d, *J* = 26.4 Hz), 44.5 (d, *J* = 20.4 Hz), 27.3 (d, *J* = 6.0 Hz), 26.1 (d, *J* = 3.6 Hz), 25.9, 25.7, 25.6; ^19^F NMR (470 MHz, CDCl_3_) δ −117.8 (m); [α]_D_^22^ = −6.2 (*c* 0.64, CHCl_3_); anal calcd (%) for C_8_H_14_ClFO: C, 53.19; H, 7.81; Cl, 19.62; F, 10.52; O, 8.86; found: C, 52.52; H, 7.88. The enantiopurity was determined after conversion into the corresponding 2-naphthoate **15e** by a procedure similar to that employed for the synthesis of **15c**. The crude mixture was purified by silica gel column chromatography (hexane/ethyl acetate = 20:1) to give 81% yield of **15e**.

**2-Chloro-2-cyclohexyl-2-fluoroethyl 2-naphthoate (15e, 96% ee)**: ^1^H NMR (500 MHz, CDCl_3_) δ 8.65 (s, 1H), 8.08 (d, *J* = 10.32 Hz, 1H), 7.99 (d, *J* = 8.41 Hz, 1H), 7.94–7.87 (m, 2H), 7.66–7.54 (m, 2 H), 4.75 (br d, *J* = 17.5 Hz, 1H), 4.75 (br d, *J* = 19.0 Hz, 1H), 2.25–2.16 (m, 1H), 2.09–2.00 (m, 1H), 1.96–1.80 (m, 3H), 1.76–1.66 (m, 1H), 1.49–1.35 (m, 1H), 1.36–1.15 (m, 4H); ^13^C NMR (100 MHz, CDCl_3_) δ 165.6, 135.7, 132.4, 131.6, 129.5, 128.6, 128.4, 127.8, 126.8, 126.5, 125.2, 116.0 (d, *J* = 248.2 Hz), 66.7 (d, *J* = 25.9 Hz), 45.4 (d, *J* = 20.1 Hz), 27.4 (d, *J* = 5.8 Hz), 26.1 (d, *J* = 2.8 Hz), 25.8, 25.7, 25.6; ^19^F NMR (470 MHz, CDCl_3_) δ −114.3 (m); [α]_D_^24^ = −13.7 (0.36, CHCl_3_); anal calcd (%) for C_19_H_20_ClFO_2_: C, 68.16; H, 6.02; Cl, 10.59; F, 5.67; O, 9.56; found: C, 68.03; H, 5.98. The enantiopurity was determined by HPLC (200:1 hexane/2-propanol; 0.5 mL/min; using a CHIRALCEL OJ-H column (0.46 cm Ø × 25 cm)): 22.5 min (major) and 25.4 min (minor).

**2-Chloro-2-fluoro-2-phenylethanol (4f, 90% ee)**: ^1^H NMR (500 MHz, CDCl_3_) δ 7.59–7.53 (m, 2H), 7.46–7.40 (m, 3H), 4.15–4.04 (m, 2H), 2.15 (t, *J* = 7.3 Hz, 1H); ^13^C NMR (125 MHz, CDCl_3_) δ 137.7 (d, *J* = 22.6 Hz), 129.8, 128.6, 125.3 (d, *J* = 7.5 Hz), 112.9 (d, *J* = 247 Hz), 70.2 (d, *J* = 26.4 Hz); ^19^F NMR (470 MHz, CDCl_3_) δ −118.2 (t, *J* = 18.8 Hz); [α]_D_ = −76.5 (*c* 0.6, CHCl_3_). The enantiopurity was determined by HPLC (99:1 hexane/2-propanol; 1 mL/min; using a CHIRALPAK IC column (0.46 cm Ø × 25 cm)): 19.1 min (major) and 21.1 min (minor).

**2-Chloro-2-fluoro-3,3-dimethylbutan-1-ol (4g) and 2-Chloro-3,3-dimethylbutan-1-ol (5g): 4g** and **5g** were inseparable by column chromatography. Therefore, isolation and determination of their enantiopurity were performed after the conversion into the corresponding 2-naphthoates **15g** and **16g** by a procedure similar to that employed for the synthesis of **15c**. The crude mixture was purified by silica gel column chromatography (hexane/CH_2_Cl_2_ = 3:1) to give 87% yield of **15g**, along with 80% yield of **16g**.

**2-Chloro-2-fluoro-3,3-dimethylbutyl 2-naphthoate (15g, 99% ee)**: ^1^H NMR (400 MHz, CDCl_3_) δ 8.68 (s, 1H), 8.12 (d, *J* = 8.9 Hz, 1H), 7.98 (d, *J* = 8.2 Hz, 1H), 7.92–7.88 (m, 2H), 7.63–7.54 (m, 2H), 4.89–4.78 (m, 2H), 1.26 (s, 9H); ^13^C NMR (125 MHz, CDCl_3_) δ 166.0, 135.7, 132.4, 131.6, 129.5, 128.5, 128.3, 127.8, 126.7, 126.6, 125.2, 119.1 (d, *J* = 251.9 Hz), 66.0 (d, *J* = 25.2 Hz), 40.8 (d, *J* = 20.4 Hz), 25.5 (d, *J* = 3.6 Hz); ^19^F NMR (470 MHz, CDCl_3_) δ −120.3 (m); [α]_D_^20^ = −22.5 (*c* 1.4, CHCl_3_); anal calcd (%) for C_17_H_18_ClFO_2_: C, 66.13; H, 5.88; found: C, 65.88; H, 6.10. The enantiopurity was determined by HPLC (200:1 hexane/2-propanol; 1.0 mL/min; using a CHIRALPAK IB-3 column (0.46 cm Ø × 25 cm)): 9.7 min (minor) and 14.0 min (major).

**2-Chloro-3,3-dimethylbutyl 2-naphthoate (16g, 29% ee)**: ^1^H NMR (500 MHz, CDCl_3_) δ 8.65 (s, 1H), 8.09 (d, *J* = 8.6 Hz, 1H), 7.98 (d, *J* = 7.6 Hz, 1H), 7.91–7.89 (m, 2H), 7.62–7.55 (m, 2H), 4.81 (dd, *J* = 3.1, 11.9 Hz, 1H), 4.45 (dd, *J* = 8.8, 11.9 Hz, 1H), 4.11 (dd, *J* = 3.1, 8.8 Hz, 1H), 1.16 (s, 9H); ^13^C NMR (125 MHz, CDCl_3_) δ 166.5, 135.6, 132.4, 131.3, 129.4, 128.3, 128.2, 127.7, 127.0, 126.7, 125.2, 70.1, 66.4, 35.2, 27.0; [α]_D_^20^ = +16.2 (*c* 1.3, CHCl_3_); anal. calcd (%) for C_17_H_19_ClO_2_: C, 70.22; H, 6.59; found: C, 69.92; H, 6.88. The enantiopurity was determined by HPLC (200:1 hexane/2-propanol; 1.0 mL/min; using a CHIRALPAK AS-H column (0.46 cm Ø × 25 cm)): 7.2 min (major) and 8.3 min (minor).

**2-Fluoro-3-(4-isopropylphenyl)-2-methylpropan-1-ol (13, 47% ee)** [7]: ^1^H NMR (500 MHz, CDCl_3_) δ 7.16 (s, 4H), 3.61–3.56 (m, 2H), 2.96 (br d, *J* = 16.5 Hz, 1H), 2.96 (br d, *J* = 20.5 Hz, 1H), 2.91–2.85 (m, 1H), 1.82 (br s, 1H), 1.27 (d, *J* = 21.8 Hz, 3H), 1.24 (d, *J* = 6.9 Hz, 6H); ^13^C NMR (125 MHz, CDCl_3_) δ 147.3, 133.2 (d, *J* = 4.8 Hz), 130.3, 126.3, 97.4 (d, *J* = 170 Hz), 67.5 (d, *J* = 22.8 Hz), 41.9 (d, *J* = 22.8 Hz), 33.7, 24.0, 20.9 (d, *J* = 22.8); ^19^F NMR (470 MHz, CDCl_3_) δ −154.7 (m); [α]_D_^25^ = −7.0 (*c* 0.60, CHCl_3_); The enantiopurity was determined by HPLC (99:1 hexane/2-propanol; 1 mL/min; using a CHIRALCEL OJ column (0.46 cm Ø × 25 cm)): 17.4 min (major) and 21.8 min (minor).

**3-(4-Isopropylphenyl)-2-methylpropan-1-ol (14, 27% ee)** [22]: ^1^H NMR (500 MHz, CDCl_3_) δ 7.15 (d, *J* = 8.0 Hz, 2H), 7.10 ( d, *J* = 8.0 Hz, 2H), 3.54 (dd, 5.0, 5.7 Hz, 1H), 3.47 (dd, *J* = 4.6, 6.1 Hz, 1H), 2.88 (m, 1H), 2.71 (dd, *J* = 6.5, 6.9 Hz, 1H), 2.41 (dd, *J* = 5.3, 8.1 Hz, 1H), 1.93 (m, 1H), 1.24 (d, *J* = 6.9 Hz, 6H), 0.92 (d, *J* = 6.5 Hz, 3H); ^13^C NMR (125 MHz, CDCl_3_) δ 146.4, 137.8, 129.0, 126.3, 67.8, 39.3, 37.8, 33.7, 24.1, 16.6; [α]_D_^25^ = −2.3 (*c* 0.15, CHCl_3_); The enantiopurity was determined by HPLC (99:1 hexane/2-propanol; 1 mL/min; using a CHIRALPAK IC-3 column (0.46 cm Ø × 25 cm)): 17.6 min (minor) and 19.9 min (major).
